# Effects on Stereopsis Under Different Lighting Conditions

**DOI:** 10.22599/bioj.496

**Published:** 2026-01-27

**Authors:** Jothi Palani, Aiswaryah Radhakrishnan, Nandhakumar Murugan, Sailesh Ravi

**Affiliations:** 1 Department of Optometry, SRM Medical College Hospital and Research Centre, Faculty of Medical and Health sciences, SRM Institute of Science and Technology, Kattankulathur, Chengelpet, Tamil Nadu, India

**Keywords:** Compact fluorescent lamp, incandescent, light-emitting diode, Randot stereo test, sodium vapour lamp

## Abstract

**Introduction:**

Stereopsis is the ability to perceive depth through binocular vision. Artificial lighting conditions play a significant role in visual performance, yet their specific effects on stereopsis remain poorly understood. This study aimed to investigate the effect of different light sources on stereopsis.

**Methodology:**

Forty young adults with normal binocular vision and stereoacuity better than 40 arcseconds were included. Participants were exposed to four lighting conditions [light-emitting diode (LED), incandescent, sodium vapour, and compact fluorescent lamp (CFL)] with illumination levels set at 400 lux. Stereoacuity was measured using the Randot stereo test under these lighting conditions, with the order of exposure randomised for each participant.

**Results:**

The median stereopsis values for CFL and LED lighting were 25 arcseconds, while those for incandescent and sodium vapour lamps were 30 arcseconds. Stereoacuity was significantly worse under sodium vapour and incandescent lighting compared to CFL and LED conditions (*p* < 0.05). However, the stereopsis values for CFL and LED did not significantly differ from the baseline (*p* > 0.05). No significant differences were found between the sodium vapour and incandescent lamps (*p* > 0.05), nor between the CFL and LED lamps (*p* > 0.05).

**Conclusion:**

Sodium vapour and incandescent lighting conditions significantly impair stereopsis, while CFL and LED lighting conditions do not adversely affect stereopsis.

## Introduction

Stereopsis represents the highest level of binocular vision (Evangelos, 2020). Stereoacuity measures the minimal horizontal disparity between images on the retina that enables the perception of depth, expressed in arcseconds (Chanchal *et al*., 2017). Stereopsis plays a crucial role in the quality of life related to vision (Chopin *et al*., 2019). Precise stereopsis is essential for daily activities, including many professions and for complex visual activities that require precise eye-hand coordination (Kulkarni, Puthran and Gagal, 2016).

Several stereoacuity tests are available, such as the Titmus, Frisby, Lang, TNO, and Randot stereo test (Deepa, Valarmathi and Benita, 2019). Among these factors, lighting plays a particularly important role, as it provides the visual input necessary for stereopsis. Inadequate or inappropriate illumination can blur vision and impair depth perception (Deepa, Valarmathi and Benita, 2019; Zhang *et al*., 2018). Although fluorescent lighting has been widely used in clinical and industrial settings, it is now being phased out in many regions, including the UK, EU, several US states, and parts of Asia, as newer technologies such as light-emitting diodes (LEDs) gain popularity. Despite this shift, sodium vapour lighting continues to be commonly used in public environments, such as street illumination, meaning that individuals are still frequently exposed to lighting sources with markedly different spectral characteristics (Hawes *et al.*, 2012). However, it has not yet fully replaced the High-Pressure Vapour Sodium (HPSV) street light that has been used throughout the country (Mirdanies, 2015).

Exposure to varying degrees of light is a common occurrence during daily activities. Due to the impact of various lighting conditions on visual functions such as visual acuity, contrast sensitivity, and stereopsis, it is crucial to consider their effects (Mosayebi *et al*., 2023). Previous studies focus on how lighting affects contrast sensitivity, colour vision and visual acuity, often overlooking stereopsis (Ram and Bhardwaj, 2018; Tidbury, Czanner and Newsham, 2016). Additionally, past findings on coloured lighting suggest that depth perception errors are smaller under white or yellow light stimuli compared to green, underscoring that lighting characteristics, beyond intensity, can influence stereopsis (Huang, 2007).

Stereopsis is routinely evaluated in clinical practice, particularly in the diagnosis and management of binocular vision anomalies, amblyopia, and prior to or following surgical interventions such as strabismus or refractive surgery. In most clinical settings, stereo tests are performed under standardised illumination, typically provided by uniform white fluorescent or LED lighting. In contrast, everyday environments expose individuals to a wide range of lighting sources – such as sodium vapour, incandescent, CFL, and LED lamps – that vary considerably in their spectral properties and intensity. Since stereo tests are also used in non-clinical settings, for example, in school screenings or workplace vision programmes, it is important to understand how such lighting variations might influence outcomes.

Beyond clinical testing, stereopsis plays a critical role in daily tasks that require fine depth judgements, including driving at night under sodium vapour streetlights, performing fine surgical or technical work, and engaging in sports. If stereoacuity is diminished under certain lighting conditions, this may not only limit the reliability of clinical assessments but could also have direct consequences for safety and performance in daily life. Therefore, this study aimed to evaluate stereopsis under different artificial lighting conditions and compare these effects with standard clinical illumination, in order to better understand both the reliability of stereo testing and the potential impact of lighting on depth perception in real-world contexts.

## Methodology

This study was conducted after obtaining approval from the Institutional Ethics Committee (Ethics Clearance Number: SRMIEC-ST0224–934) and adhered to the tenets of the Declaration of Helsinki. This prospective, experimental, cross-sectional study was conducted at the Medical College. A total of 40 participants (18 males, 22 females) aged 17–25 years were recruited using a convenience sampling method. Inclusion criteria were normal binocular vision with stereoacuity of 40 arcseconds or better and visual acuity of 20/30 or better in each eye. Participants were excluded if they had a history of ocular disease, trauma, surgery, systemic conditions affecting vision, or non-strabismic binocular vision anomalies (NSBVA).

Written informed consent was obtained from all participants prior to testing. Each underwent a comprehensive ocular examination to confirm normal (or corrected-to-normal) vision and ocular health. The examination included detailed case history, evaluation of distance visual acuity by Snellen chart at 6 m and near visual acuity by using continuous reading N-Notation chart, measurement of the refractive status of the eye objectively through retinoscope and subjectively by using standard defogging procedures, evaluation of the ocular muscle imbalance was done through extraocular motility test and cover test, evaluation of pupillary response was done by swinging flash light test, anterior segment evaluation using slit lamp biomicroscopic and retinal evaluation using 90D lens with fundus biomicroscopic technique. Participants further underwent screening for NSBVA. It included stereopsis, near point of accommodation, near point of convergence, negative and positive relative accommodation, accommodation facility, vergence facility, negative and positive fusional vergence, monocular estimation method retinoscopy and AC/A ratio. Only optometry students who had not previously performed stereopsis measurements were included, in order to minimise training-related bias. Students from different academic departments were recruited to ensure diversity.

**Figure 1:**
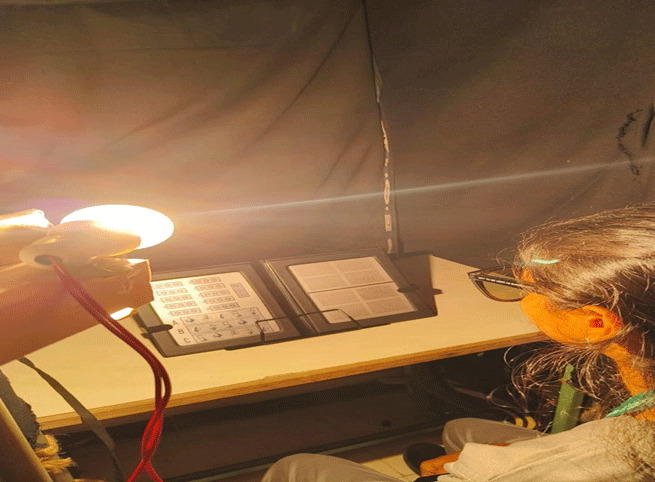
Experimental setup.

The baseline stereopsis was measured using Randot stereo test, under standard clinical illumination of 400 lux. Participants wore the polaroid glasses provided with the Randot stereo test during all measurements. Each participant was exposed to four lighting conditions – LED, incandescent, sodium vapour, and CFL – in separate sessions. The order of exposure was randomised for each participant. Testing was conducted in a darkened room, with only the procedure area illuminated at approximately 400 lux and the surrounding surfaces covered with opaque material to avoid exposure to outside light. For each lighting condition, participants were exposed for approximately 5 min before testing. Then the stereoacuity was measured using the Randot Stereo Test. Sessions for different lighting conditions were held on separate days, with a minimum washout period of three days. The procedure table measured 47 × 75 cm. A brightness level of 400 lux was chosen for illumination, and this was achieved by adjusting the height of each lamp. Specifically, the 100 W incandescent lamp (yellow, CCT –3000K) was positioned 44 cm from the table surface, the 9 W LED lamp (white, CCT –6500K) at 41 cm, the 70 W sodium vapour lamp (Golden yellow, CCT –2000K) at 50 cm, and the 12 W compact fluorescent lamp (white, CCT –6500K) at 40 cm. Illumination at the test plane was verified prior to each measurement using a calibrated lux metre. The Randot stereo test was placed on a reading stand at 40 cm. All light sources were positioned behind the participant’s head and directed forwards and downward at approximately 45°, to ensure uniform illumination of the test surface while avoiding direct glare into the eyes. The overall experimental arrangement, including the participants’ seating, the reading stand, the test booklet, and the light sources are illustrated in Figure 1.

Data were entered into Microsoft Excel and analysed using SPSS software (version 20). Descriptive statistics (median and interquartile range) were calculated for stereopsis under each lighting condition. Data normality was assessed using the Shapiro–Wilk test. And as the data were non-normally distributed, the Friedman test was used to compare stereopsis across the four lighting conditions. Post-hoc pairwise comparisons were conducted to identify specific differences (significance value: *p* < 0.05). Spearman’s correlation analysis was used to examine the association between baseline stereopsis and changes under each lighting condition. The Kruskal–Walli’s test evaluated the distribution of stereopsis changes across baseline stereopsis groups, and the Mann–Whitney U test compared stereopsis scores between male and female participants for each lighting condition.

## Results

**Figure 2:**
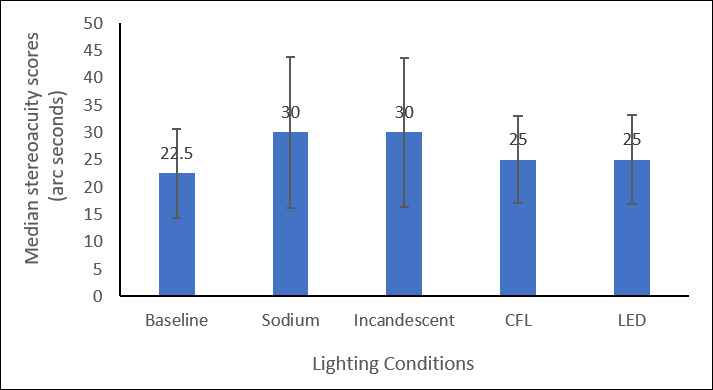
Stereopsis score under different lighting conditions.

A total of 40 participants, with a median age of 19 ± 2 years, were included in the study. The Shapiro–Wilk test indicated that stereopsis values under sodium vapour lamp conditions did not significantly deviate from a normal distribution (*p* = 0.637). The median baseline stereopsis under clinical standard illumination was 22.5 ± 10 arcseconds, under different lighting conditions, the median stereopsis under sodium vapour lamp was 30 ± 14 arcseconds, incandescent lamp was 30 ± 15 arcseconds, CFL was 25 ± 10 arcseconds, and LED lamp was 25 ± 18 arcseconds. A Friedman test revealed significant differences in stereopsis across the four lighting conditions (χ² = 73.774, *p* < 0.001). Post-hoc pairwise comparisons with Bonferroni correction showed that stereopsis was significantly reduced under sodium vapour (*p* < 0.001) and incandescent lighting (*p* = 0.003) compared with baseline. In contrast, stereopsis under CFL (*p* = 1.000) and LED (*p* = 1.000) did not differ significantly from baseline. No significant differences were found between sodium vapour and incandescent lamps (*p* = 1.000), but both showed significantly poorer stereopsis than CFL and LED lighting (all *p* < 0.05). No significant differences were observed between CFL and LED lamps (*p* = 1.000). These findings are illustrated in Figure 2, which shows the median stereoacuity scores across the lighting conditions.

Spearman’s correlation analysis showed that baseline stereopsis was not significantly associated with stereopsis changes under any lighting condition: sodium vapour (*r* = 0.052, *p* = 0.751), incandescent (*r* = –0.001, *p* = 0.995), CFL (*r* = –0.266, *p* = 0.097), or LED (*r* = –0.266, *p* = 0.097). However, changes in stereopsis under sodium vapour and incandescent lighting were significantly correlated (*r* = 0.439, *p* = 0.005), as were changes under CFL and LED lighting (*r* = 0.621, *p* < 0.001), indicating that these paired lighting types produced similar stereopsis effects.

The Kruskal–Walli’s test revealed no statistically significant differences in stereopsis changes across baseline groups under sodium vapour (*p* = 0.212), incandescent (*p* = 0.801), CFL (*p* = 0.368), or LED (*p* = 0.368), suggesting that lighting effects were consistent regardless of baseline stereopsis level.

Finally, Mann–Whitney U tests showed no significant gender differences for stereopsis under any condition: baseline (*U* = 161.500, *p* = 0.284), sodium vapour (*U* = 161.500, *p* = 0.307), incandescent (*U* = 168.000, *p* = 0.409), CFL (*U* = 152.000, *p* = 0.188), or LED (*U* = 147.500, *p* = 0.147). These findings indicate that stereopsis was not influenced by gender across lighting conditions.

## Discussion

This study investigated the effects of different lighting conditions on stereopsis in young emmetropic adults. The main finding was that stereopsis was significantly reduced under sodium vapour and the incandescent lamp compared with baseline, whereas CFL and LED lighting-maintained performance similar to the baseline levels. These results suggest that certain artificial light sources can adversely affect fine depth perception, while others maintain visual performance. Although the differences were statistically significant, the magnitude of change was relatively small (≈7–8 arcseconds). Whether this difference is clinically meaningful warrants careful consideration. For most everyday visual tasks, small shifts of this size are unlikely to cause noticeable impairment, as stereoacuity thresholds of 40 arcseconds or better are generally considered adequate for functional depth perception. However, in visually demanding contexts – such as microsurgery, precision engineering, sports, or night-time driving – even subtle reductions in stereopsis could contribute to errors or accidents. Thus, while the group-level effect may not imply major clinical consequences for all individuals, the potential performance implications in high-precision tasks should not be overlooked.

Our findings that stereopsis is reduced under sodium vapour and incandescent lighting are in line with earlier evidence showing that ambient illumination can significantly affect perceived depth quality. For instance, Pölönen *et al*. (2011) reported that changes in ambient illumination levels influenced both the quality of autostereoscopic displays and depth perception. This supports the notion that stereopsis is sensitive not only to binocular visual mechanisms but also to external lighting environments.

These findings align with the earlier research by Varadharajan *et al*. (2009), who reported no reduction in stereopsis under CFL and LED lighting conditions. Similarly, Radhakrishnan *et al*. (2023) found higher error rates in visual tasks under incandescent lighting compared with CFL Our study extends this evidence by quantifying the stereoacuity changes directly, thereby confirming that sodium vapour and incandescent lighting impair depth perception compared to CFL and LED conditions.

Interestingly, baseline stereopsis did not predict how participants responded to lighting changes, suggesting that the effect of light type is fairly uniform across people with different starting levels of stereoacuity. The correlations observed between sodium vapour and incandescent, and between CFL and LED, indicate that these pairs of lighting types tend to influence stereopsis in similar ways. Gender differences were not found, which supports the generalisability of the results.

While this study provides valuable insights into the effects of different illumination conditions on stereopsis, several limitations should be acknowledged. The study included only young adults with normal vision, so the results may differ in individuals with refractive errors, binocular vision anomalies, or in older populations. Moreover, although the findings were statistically significant, the effect size was modest; further research is needed to determine whether such changes have measurable impacts on real-world performance. Exploring how different lighting conditions influence both general daily activities and specialised professional tasks could provide more practical insights. Finally, future work should examine a wider variety of light sources, including newer technologies and varying illumination intensities, to better understand how spectral quality and brightness influence depth perception.

## Conclusion

This study found that stereopsis significantly worsened under sodium vapour and incandescent lighting compared to LED and CFL lamps. No significant differences in stereopsis were observed between LED and CFL lighting, suggesting that these modern light sources have a lesser impact on stereopsis. This study provides valuable insights into how various artificial lighting conditions can affect stereopsis, highlighting the need for careful selection of lighting in settings where optimal visual performance is essential.

## Data Accessibility Statement

The data that support the findings of this study are available from the corresponding author upon reasonable request.
